# EUROPA: A Case Study for Teaching Sensors, Data Acquisition and Robotics via a ROS-Based Educational Robot

**DOI:** 10.3390/s20092469

**Published:** 2020-04-27

**Authors:** Georgios Karalekas, Stavros Vologiannidis, John Kalomiros

**Affiliations:** Department of Computer, Informatics and Telecommunications Engineering, International Hellenic University, 62124 Serres, Greece

**Keywords:** educational robotics, data acquisition, sensors, ROS, STEM

## Abstract

Robots have become a popular educational tool in secondary education, introducing scientific, technological, engineering and mathematical concepts to students all around the globe. In this paper EUROPA, an extensible, open software and open hardware robotic platform is presented focusing on teaching physics, sensors, data acquisition and robotics. EUROPA’s software infrastructure is based οn Robot Operating System (ROS). It includes easy to use interfaces for robot control and interaction with users and thus can easily be incorporated in Science, Technology, Engineering and Mathematics (STEM) and robotics classes. EUROPA was designed taking into account current trends in educational robotics. An overview of widespread robotic platforms is presented, documenting several critical parameters of interest such as their architecture, sensors, actuators and controllers, their approximate cost, etc. Finally, an introductory STEM curriculum developed for EUROPA and applied in a class of high school students is presented.

## 1. Introduction

Robotics represents an innovative field that encompasses various scientific domains, from physics and electronics to mechanical engineering, mathematics and computer programming. The vast field of artificial intelligence is also relevant to autonomous robots. Educational robotics is a rapidly evolving multidisciplinary domain that brings together educators, companies and researchers in an effort to create a new learning environment in schools and universities. Rooted in Papert’s seminal ideas on computational thinking using LOGO programming and Mindstorms [1], educational robotics is increasingly becoming popular in the classroom. It is supportive in teaching Science, Technology, Engineering and Mathematics (STEM) [2,3] and in some cases it transcends the traditional science border and becomes supportive of artistic activities (STEAM) [4,5]. 

Introducing robots in the classroom can become a suitable tool to instill new skills in young learners and students; besides teamwork and creativity, students can develop interest in practical concepts in physics and mathematics and get acquainted with topics in engineering [6]. Robotics can help teachers present the concept of system engineering and control. In addition, it can motivate young students towards STEM-oriented career paths, which has become important following the expansion of technology markets and their demand for engineering graduates. Interacting with robots can even be beneficial for children in a kindergarten [7,8] and it can play an important therapeutic role in special education [9].

Innovative learning based on robotics also brings about the need to develop new curricula for schools and universities, to cover gaps in documentation for teachers and students and to develop new products in the form of simple, low-cost mobile platforms, according to the educational level of the target group. On the other hand, introducing robotics courses in schools calls for the parallel development of methods for the assessment of new educational technologies and the dissemination of their results [10]. As robot-based technologies are becoming mainstream in schools and universities, educational robotics is gaining its own spin and status among researchers, markets and educators, with new emerging conferences [11], special issues [12] and products [13,14] and with a boost in the relevant literature [15].

Within the constructivist approach of Piaget [16] and Papert [17], constructing the robot can be considered an integral part of the learning procedure. Construction not only stimulates the creativity and enthusiasm of young learners through an open-ended, problem-solving process in the real world, but it also instills technological literacy and better understanding of the different parts that make up a robot as an engineering system. This is especially true for primary and secondary (K-12) education [18]; however, it can also find application in college or university education, where lab exercises on robotics often include a basic assembly of a simple robot, like a mobile cart driven differentially. Several of the educational bots currently available as market products allow some level of assembly of the robot from parts, while others encourage extensions of a ready product.

The main challenges when designing a new robotic platform for education are component accessibility, flexibility and cost. It is preferable to design platforms based on commodity components that can be easily accessed in the market and replaced when needed. The platform should be flexible enough to adjust to different teaching scenarios. In part, this means that an educational robot should best follow a modular architecture in terms of sensors and accessories and in terms of software, especially in order to span different curricula. Finally, a low-cost platform makes an investment in robotic technology more plausible for a large classroom, where each small group of three or four students should share a robot with its accessories and build several projects around it.

Using open-source software and open hardware in designing an educational system is important, especially for high school secondary education and for university courses. Open hardware, like Arduino Uno [19], with its free programming environment and community support [20] can increase the level of student creativity and engagement in a robotics project. Similarly, the Raspberry Pi [21], although it does not exactly represent open hardware, is supported by a large community, runs a version of the Linux Operating System and can be programmed using Python. Python is widely taught in Informatics lessons in various high school curricula, as is the case in Greece. Hardware boards like the above provide user-friendly input/output support and can be easily adopted for other technology-oriented extra-curricular activities, beside educational robotics.

The power of open software in educational robotics is best exemplified by the Robot Operating System (ROS). ROS [22] is a middleware that runs on Linux and recently on Windows 10 and has become a standard for robotics, in industry, education and research. It provides easy access to complex software components and communicates with a great variety of hardware, like sensors and actuators. It allows the robot integration with tools for simulation and visualization [23,24,25] and with libraries for robotic vision, artificial intelligence and Simultaneous Localization and Mapping (SLAM). These powerful functionalities transform the robot from a simple programmable automatic system to a true autonomous intelligent device, compliant with the technology of the Internet of Things (IoT).

A number of educational platforms have been presented as market products and have been introduced in various levels of education. From Beebot [26,27] to Thymio II [28,29] to Scribbler 3 [30] and LEGO EV3 [31,32,33], the educational market has provided teachers with ingenious tools to devise innovative lessons on almost everything. Activities range from exhibiting a practical algorithm in kindergarten to teaching concepts on motion and automation to understanding basic programming and the role of sensors and actuators in a control loop. More advanced platforms, like the epuck [33,34], the Turtlebot [35] and the Duckietown [36], introduce students to the use of single board computers, path planning and environmental mapping. They use cameras and artificial intelligence for object recognition and are suitable for research on advanced stochastic algorithms for localization and mapping. More industrial-like robotic platforms, like DaNI and VEGA, are often adopted for the needs of the postgraduate level and for research [37]. In the same category, the Pioneer mobile platforms by Adept have been very popular for autonomous navigation research but they are gradually replaced by a line of ROS based autonomous mobile platforms, like the Leo Rover [38]. Finally, pure industrial grade robot platforms, like the Robotnik^TM^ Summit-XL [39] or the Husky and Jackal unmanned mobile bases by Clearpath Robotics^TM^ [40], are fully ROS based customizable platforms, suitable for research projects and industrial or agricultural applications.

The contribution of this paper is twofold: first, we present a comprehensive review of the state of the art on educational robotic platforms through K12 to college and university and second, we present EUROPA, a new educational mobile platform based on ROS, which has been developed following the main guidelines stipulated above: constructivist approach, accessibility of parts, modular flexibility and open hardware and software technology. The platform has been introduced in a secondary school class following the Greek educational system and has been positively assessed by students and tutors. A short curriculum is also proposed for the blending of robotic technology with STEM teaching, in secondary school.

The rest of the paper is structured as follows. In Section 2 we present a comprehensive state-of-the-art review on the technology of educational mobile platforms through various levels of education. In Section 3 the hardware and software architecture of the proposed EUROPA platform with its ROS software architecture is presented. In Section 4 EUROPA is studied as a paradigm of introducing a robot in class and the assessed curriculum is outlined. A configuration of EUROPA for teaching more advanced robotics is also proposed, aiming to lessons on autonomous driving, typically applying to university education. Finally, Section 5 concludes the paper.

## 2. State-of-the-Art Educational Platforms

In this section, several well-known mobile platforms used in education are presented, starting with bots that have been adopted to teach computational thinking and basic notions of programming in elementary school, proceeding with platforms that can be used in STEM classes which enhance engineering literacy in high school and ending with projects designed to teach autonomy or test computer vision and navigation algorithms in university and research. Of course, this presentation cannot be exhaustive, since there is a large number of products, some very successful, others very promising, several of low cost and some based on open hardware/software. However, we took care to include those platforms that appear often in the literature on educational robotic technology or are promising in our opinion to lead a trend in a specific educational level. A comparative examination of the technology and specifications of such successful platforms can indicate how the next generation of educational robotic technology is going to evolve. A reference to most of the products that are not directly presented in this section can be found in the proposed literature. Humanoids and torsos, like NAO [41], Pepper [42] or the Robotis OP3 [43] are becoming part of the educational robotics ecosystem; however, this review is limited to wheeled mobile platforms with a relatively low degree of complexity and with affordable cost in the context of school/college education.

**Figure 1:**
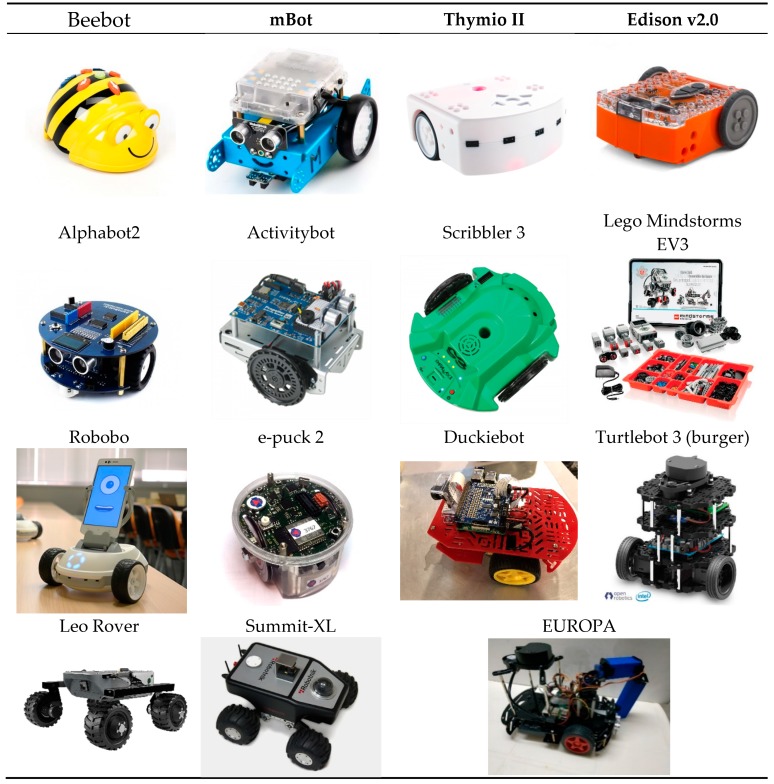
Images of the mobile robot platforms listed in Table 1.

Table 1 lists fourteen widely used educational platforms as well as our proposed EUROPA robot. The table presents the basic technologies supported by each platform and the level of education they best fit in. The current approximate cost of the platform is also given, as it is suggested by the distributor. In the last column, a reference to the literature presenting the platform capabilities or its exemplary use in class is provided. Each one of the listed systems is illustrated in Figure 1. One industrial platform, the Summit-XL, is also presented as a comparative reference.

The Beebot represents a category of toy robots appropriate for teaching introductory notions of control. It illustrates directional language and following steps in problem solving, like in a maze. It is used widely in kindergarten and elementary education with exciting results [26]. Being a toy rather than a well-defined robot vehicle, it is not well documented with regard to its mechanical and electronic specifications.

The mBot is an introductory small robot by Makeblock [44]. It is based on a light metal chassis and can be assembled from parts. It can be programmed either by a block-based graphical programming interface based on scratch or using the Arduino Integrated Development Environment (IDE). Makeblock provides STEM teaching case studies in its webpage.

Thymio II is a versatile open platform suitable for all levels of K12 education, best documented with activities for elementary school. It supports six basic “behaviors”, allowing obstacle avoidance, line following, hand following, etc [28]. It is based on a PIC24 microcontroller unit with an H-bridge for motor driver. It features a number of sensors, like accelerometer, thermometer and infrared proximity sensors for obstacle avoidance. Its basic actuators are two basic motors driven differentially, a loud-speaker and leds. The platform is expandable using accessories and is poised to evolve into a STEM teaching tool for higher grades or possibly into a ROS platform [45].

Edison is primarily a very affordable mobile platform for teaching STEM [46]. It is equipped with a similar range of sensors and actuators, like Thymio, although it is not as “moody” and easy to personalize as Thymio and it does not belong to the open hardware and software camp. It can avoid obstacles and track a line using IR sensors and can respond to sound or play music using the integrated sound/buzzer module. It can interact with other robots using light signals. It can be programmed using three different versions of a programming environment: EdBlocks for programming with icons, EdScratch, using a block based visual programming style and the text based EdPy, which is a version of the Python language.

Lego Mindstorms EV3 [32] is a kit for educational robotics, consisting of a programmable brick and a set of motors, sensors and TECHNIC elements that can be used to assemble the robot. EV3 continues the line of Mindstorms NXT, featuring a more powerful ARM9 processor and 64MB RAM. It supports Wi-Fi and Bluetooth connectivity and can be programmed using the custom programming environment Lego Mindstorms EV3 Home Edition, which is based on a block-based graphic language originating from LabVIEW, by National Instruments. This platform is widely used in competitions. 

Alphabot2 is a small mobile platform by Waveshare [47] that comes in various flavors. In its cost-effective version it hosts an Arduino controller, while it can also come with a Raspberry Pi or with a BBC micro:bit microcontroller. An ultrasonic distance sensor is used in all variations for obstacle avoidance. Alphabot2 represents open hardware and can be programmed using the Arduino IDE or Python scripts, depending on the controller.

Scribbler 3 and Activitybot are robots powered by the well-known Propeller CPU made by Parallax [30]. Scribbler 3 is a robust plastic platform suitable mostly for elementary education, which can be programmed using a block-based programming language. Activitybot features a metallic chassis and a small breadboard for adding sensors and other circuitry. Besides the block-based graphical environment, Activitybot can also be programmed in C. 

The e-puck 2 [33,34] is a small differential wheeled robot designed for research and education. It is powered by a STM32F4 microcontroller and features many sensors, like IR and Time of Flight distance sensor, IMU, color sensor, etc. It is also suitable to study swarm and evolutionary robotics. It supports C programming and ROS libraries.

The Robobo [48,49] is different from the above and represents an interesting experiment by the University of Coruña. It consists of a mobile base and an attached smartphone. It makes use of the CPU power of the smartphone and of sensors incorporated in it, mainly cameras, gyroscope, accelerometer and GPS. The robot can be programmed using a Scratch web-based editor or a text-based language and aims to introduce lessons on autonomy to secondary school students [50].

The Turtlebot 3 [35] is a relatively low-priced, small size differentially driven mobile platform based on ROS. It is an open source collaboration project by several partners [14] and it is assembled from high quality modular parts. It is based on 3D-printed expandable chassis and is controlled by an effective controller and Single Board Computer. The main sensor of the Turtlebot is a low-cost LIDAR that is able to perform navigation tasks and SLAM. It can also be expanded by other sensors, like RGB and RGBD camera, supported by ROS software modules. It can be used as a mobile manipulator, by attaching a manipulator module. The Turtlebot has been used successfully in graduate education and research [52].

The Duckietown [36] is an open project proposed by a MIT team, intended for teaching robot autonomy or individual aspects of autonomous driving, like vision or nonlinear control, at a graduate or postgraduate level. It consists of the Duckiebots, which are open inexpensive differentially driven mobile bots and a model environment representing a miniature town with roads, signs and inhabitants, assembled from modular tiles. The sole sensor of the Duckiebot is a monocular camera. Vision based algorithms are responsible for lane detection, sign or object recognition and localization of the robot in the Duckietown [13]. More advanced algorithms allow path planning using metric and topological maps as well as vision-based Simultaneous Localization and Mapping. The system supports ROS for data transfer between software nodes. It can be expanded for the study of multirobot behavior. The cost given in Table 1 refers to a single bot without the Duckietown.

The Leo Rover [38] is a robust open source platform designed for autonomy research in outdoor environment. It is customizable by add-ons, like a manipulator, GPS module, camera, IMU, etc. The robot is driven by four independent DC gear motors with suspension system and it is powered by Raspberry Pi and a Core 2 ROS driver board. Although it represents an open platform with a GitHub repository, it requires extensive programming by the developer for the execution of every specific task. Therefore, its scope is different than that of educational boards.

Finally, the Summit-XL platform by Robotnik [39] is a versatile strong frame, based on a four-wheel skid-steering configuration, designed for high load capacity. It can be easily switched to an omni-directional configuration using mecanum wheels. It features an IMU and can receive a camera and a laser scanner. It also features a default radio system for remote operation and is suitable for research and surveillance. It is controlled by a PC and it is programmed with open ROS architecture. Robotnik produces a line of industrial-grade robots, of which the Summit-XL is a midrange example.

Beside the platforms of Table 1, a reference should be given to a slightly different flavor of educational solutions, namely the kits by Vex Robotics [53] and Pitsco/Tetrix Robotics [54]. These kits provide robust metal parts, sensors, motors, electronics and other hardware for the assembly of a range of robots for education, hobby and competitions. They represent an advanced constructivist approach, with an average cost of a medium range kit of the order of 900 €. 

The platforms presented above give a review of current educational robotic technology and trace its future evolution. Table 1 reveals a gap in low-cost educational platforms based on ROS. However, a unifying middleware like ROS is imperative for flexibility, adaptability, ease of development and community support. In addition, the above analysis shows that connectivity within a local computer network and browser-based programming tools are definite trends. Finally, the success of educational platforms depends on their low-cost and on the versatility of programming tools, from block-based to text-based programming, covering different educational levels and needs. These virtues were exploited in the design and implementation of the EUROPA platform. 

## 3. Materials and Methods: Presentation of the EUROPA Platform

### 3.1. Overview of EUROPA 

**Figure 2:**
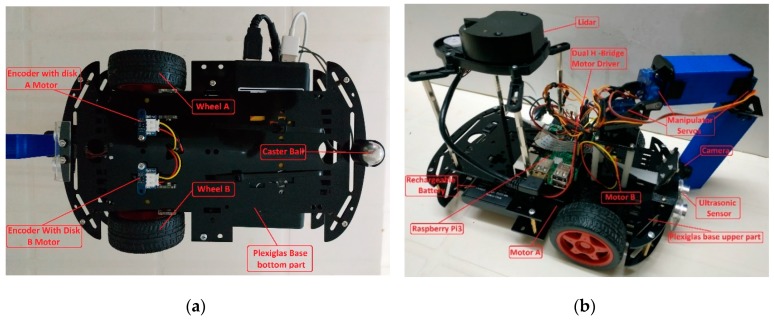
EUROPA and its components; (**a**) Bottom view; (**b**) Side view.

EUROPA (EdUcational Ros rObot PlAtform) is a two-wheel, inexpensive differential drive robot with a manipulator. It is adequately scalable and flexible to fit into different educational levels and different curricula. It allows programming with introductory or more advanced tools, depending on educational level. Its main controller is the Raspberry Pi 3 B+. An introductory presentation of the initial version of EUROPA was given in [51]. Figure 2 shows the basic EUROPA components.

EUROPA follows the open hardware paradigm and uses open source software. The robot can be built by the students themselves, under the appropriate instructions from their teachers, providing an opportunity for hands-on experience with principles of electricity, electronics and engineering. Although the robot can be used for Science, Technology, Mechanics and Mathematics (STEM) [2,3], it can also be upgraded with sensors like a LIDAR, to allow for more advanced lessons and research on robotics. EUROPA is based on ROS, which provides interoperability and extensibility. Although ROS stands for Robot Operating System, it is really a framework that sits on top of an existing operating system such as GNU/Linux. EUROPA includes a camera that can be used for image processing and object recognition. In addition, it supports a plethora of sensors that can be added to the Raspberry Pi board in order to support user-defined tasks. 

EUROPA includes a simulation environment. The robot was described in Unified Robot Description Format (URDF) and is simulated in the Gazebo environment [23]. Robot simulation allows children to easily and safely experiment with algorithms and develop skills related to computer programming. Following simulation, students will be able to choose the best performing algorithms, test them on the real robot in the physical world and understand the differences between robot behavior in a simulated as opposed to a real environment. In addition, using the interface of rviz [25], the popular visualization tool for ROS, they will be able to visualize depictions of the robot movement and easily control the robotic arm. Finally, they can learn concepts like odometry and sensor visualization.

### 3.2. EUROPA Hardware

EUROPA is built on a double plexiglas base, which supports all the robot’s mechanical and electronic components. A rechargeable 10000 mAh battery is included, providing power to the Raspberry Pi and motors. Two differentially driven DC motors with wheels and encoder disks are responsible for EUROPA’s locomotion, allowing a speed of up to 2 m/s with 8 N cm of maximum torque. This is enough for climbing on small ramps. In addition to the wheels, the robot rests on an omnidirectional caster ball, located on the back.

On the upper side, we find the Raspberry Pi 3 B+ board, a two-motor controller shield dual H-bridge motor driver DRV8833 [55], the Raspberry Pi Camera Module Night Vision-Adjustable Focus (5MP, 1080p) [56] and the robotic arm. The arm rests on a base made of 4 spacers 5 cm long screwed directly onto the robot chassis. The two axes of the arm are 3D printed and the joints are two Mini Pan-Tilt Kits powered by micro servo motors (Servo Micro plastic gears Feetech FS90, 1.5 kg·cm). The whole construction is characterized by simplicity and ease of assembly.

The University Edition of EUROPA features a laser scanner for 360 degrees distance measurement (LIDAR LDS1.5 [57]). It can measure a cloud of data around the robot up to a distance of 3.5 m and can support experiments on Simultaneous Localization and Mapping (SLAM).

At the bottom of the chassis there are two encoders with led-photodiode pairs (Waveshare, 12225) [58] used for odometry measurements. Finally, there is a distance meter on the front of the robot that can be used for obstacle avoidance (Ultrasonic Sensor 2–400 cm SR04) [59]. All accessories are connected directly to the Raspberry board without the need for extra electronic controllers. Table 2 presents the parts list and their approximate costs. 

### 3.3. EUROPA Software

**Figure 3:**
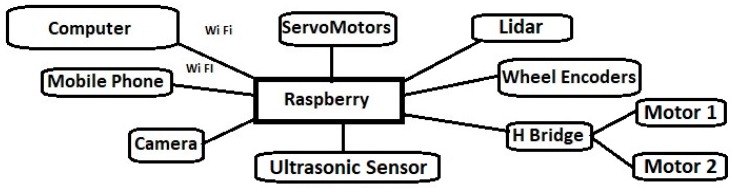
EUROPA control structure.

EUROPA uses ROS infrastructure for communication and control, as shown in Figure 3. Raspberry controls the motors and is in charge of data collection from sensors and the camera. All the drivers responsible for the control of the two DC motors, the servo motors, the ultrasonic sensor and the LIDAR are installed on Raspberry Pi. The Raspberry Pi hosts several Python scripts that act as ROS nodes. For example, they collect video from the camera [60], receive input from the LIDAR [61], measure wheel movement via wheel encoders to calculate odometry and publish the data as ROS topics. A desktop computer which is running the ROS master is connected to the robot via Wi-Fi. Using the computer, the student or teacher can run either Python scripts or ROS user interfaces (UIs) [62] to control the movement of the robot and visualize data. The robotic arm can also be controlled via rviz or RQT [24,25] from the computer. Additionally, the robot can be controlled by a mobile phone, using ROS Control API without the need of a computer. Although the proposed way is to work with the robot from a remote computer, the student or instructor can also connect a screen and a keyboard directly to the Raspberry Pi and control the robot without the need of any additional device. The rviz-based user interface can also show live video from the camera and data from the ultrasonic sensor. 

Additionally, the stream from the camera can be used to devise solutions to problems such as line following, while LIDAR and odometry can be used for Simultaneous Localization and Mapping (SLAM) [63] and navigation.

ROS and Gazebo provide communication, simulation and visualization tools. These modules are needed to perform tasks such as image processing and sensor calibration. Robot Operating System allows the use of modules and applications available in the ROS ecosystem. ROS provides modules for navigation, arm manipulation and SLAM. The ROS master is running on the PC, which is responsible for the communication between the various nodes running on the robot and computer. Any nodes that run in the ROS cluster can communicate with each other by exchanging information. Information circulates in the form of messages organized in topics, to which each one node either publishes or subscribes. 

The different nodes that exist in EUROPA robot are described below. All nodes referred to as custom nodes have been created by the EUROPA team for use with the EUROPA robot.

#### 3.3.1. Nodes that Run Exclusively on the Robot

- A custom Python node for DC motor control.
- A custom Python node for translating position information to appropriate command signals for the servo motors of the robotic arm.
- A node for streaming video from the camera.
- A node for the LIDAR operation which publishes data using ROS hls_lfcd_lds_driver
- driver [61].
- A custom Python node for publishing distance measurements collected from the distance sensor.
- A custom Python node for publishing odometry data from the wheel encoders.

#### 3.3.2. Nodes that Run either on the Remote Computer or on the Robot itself (If It is Connected to a Screen and a Keyboard)

- A custom Python node for moving the robot using the keyboard.
- A custom Python node for the movement of the robotic arm.
- A custom node to watch the video captured by the robot camera
- A custom Python node to identify color lines and to send velocity messages that control the movement of the robot.
- A custom Python node for moving the robot to a specific position on the xy plane.

#### 3.3.3. Nodes and Simulations that Run Exclusively on the Remote Computer

- Simulation of the robot in a virtual environment via the Gazebo application.
- Visualization of the robot movements and odometry via rviz.
- Control of the movements of the robotic arm through Moveit [64].
- A node responsible for SLAM using ROS’s hector_slam [65] metapackage.

## 4. Results and Discussion: EUROPA in the Real World

### 4.1. Europa in Secondary Education

Most approaches to school robotics are currently focused on writing a script of code for robot control, along with a Lego-type construction. Usually, students do not go deeper into hardware and seldom do they go properly into software design concepts. The complexity of issues like motor control, wheel encoders and other sensors is usually hidden even from the interested student. One goal of the EUROPA project is to provide the students with an open platform for mechatronics concepts, ranging from introductory to advanced. The teacher can choose to present a high-level overview of the system or to teach in depth concepts. The students can acquire hands on experience with experiments in physics, electricity and robotics. EUROPA was tested in two Greek schools, during the first semester of the school year 2019-20. A STEM curriculum with applications in sciences, engineering and programming was designed and implemented. The target group was second-grade high school students, in the Greek system, which is equivalent to tenth or eleventh grade in the K12 system (ages 16–17). The curriculum that was used is briefly described below. 

#### 4.1.1. Robot Construction

The robot was constructed by the students with instructions from the teacher, and at the same time, an introductory lesson on sensors and motors was given. Initially, there was a reference to voltage, current and operation of DC motors. Past school lessons on these topics were revisited.

#### 4.1.2. Motors and Sensors

The next step was to provide students with a basic understanding of the role of sensors and actuators. A presentation was given on servo motors and Pulse Width Modulation (PWM) was explained. A lesson on sensors was given, and different sensors were presented. The principle of data acquisition in a digital system was introduced and the use of a library for transferring data from a sensor to a Python program was explained. Then, the principle of the distance sensor was illustrated using a simple setup with a speaker and a microphone. Students were asked to calculate distance using time of flight, revisiting first grade physics. The photo-interrupter included in EUROPA provided the opportunity to introduce aspects of the interaction of light with matter. Finally, the camera was introduced and a reference to image processing was made. The role of the camera in the recognition of the environment was discussed.

#### 4.1.3. Robot Simulation

In the next lesson, the robot’s simulation was presented to students using rviz [37] and Gazebo environments. The students were also given the Unified Robot Description Format (URDF) file describing the robot. The XML file was analyzed focusing on specific physical properties of the robot. The students understood how a robot can be described using geometric figures and physical properties. Then, the students experimented by changing specific parameters to the existing robot description and saw how the robot was affected in the virtual environment. 

#### 4.1.4. Writing Python Scripts for EUROPA (Part 1)

The next lesson presented a Python script that receives input from the computer keyboard and translates it into robot motion commands. The students applied knowledge from lessons on circular motion and revisited notions on angular and linear velocity, applying them in real-world conditions. 

#### 4.1.5. Writing Python Scripts for EUROPA (Part 2)

The next lesson was to direct the robot to a specific position by applying the Pythagorean Theorem and other basic trigonometric equations. A Python script was created and explained before execution. At this point, it is important to note that students were watching the robot movements both in the simulation environment and in real life. 

#### 4.1.6. Data Acquisition from Wheel Encoders and Odometry Computation

During this lesson, the students first learned to use interrupts in order to get the encoder data and thus calculate angular velocity of each wheel. Additionally, students calculated odometry by applying high school grade physics kinematics and published odometry information to ROS.

#### 4.1.7. Controlling the Robot Arm of EUROPA (Part 1)

The next lesson focused on the robotic arm of the robot. Geometry and algebra were linked to the movement of the arm. In addition, students were introduced to the concept of torque and they were familiarized with it by using different gears in Lego constructions. Continuing with this lesson, a simple movement of the arm was performed. A Python script for arm control was provided, and the students were asked to parameterize it. In addition, they used rviz with RQT to control the robotic arm. Different angles for the servomotors were given, and the students tried to determine theoretically the position of the tip of the robotic arm. 

#### 4.1.8. Controlling the Robot Arm of EUROPA (Part 2)

Next, the students were asked to calculate the angles of the servo motors of the robotic arm in order to place the tip at a specific position in 3D space. In this way, they were introduced to the importance and difficulty of the inverse kinematic problem. When they understood the difficulty of the problem, the Moveit! package [64] was presented, which provides the arm with the capability to perform complex movements using ready-made libraries and kinematic model solutions. Again, the students had the opportunity to see the simulated and real robot repeating the same movements.

### 4.2. Advanced Robotics Course with EUROPA

With the addition of the camera and the LIDAR, EUROPA becomes an efficient platform for teaching more advanced robotics courses. Such courses are often part of the curriculum in college or university; however, interested high school students can be benefited as well. After a series of introductory notions, students can continue the learning process, focusing on concepts related to computer vision, machine learning and robot autonomy. The following experiments were demonstrated in the same class of high school students who attended the set of lessons outlined in paragraph 4.1.

#### 4.2.1. Tele-Operation of EUROPA Using the Camera

This project includes tele-operation of the robot using the camera and the distance meter. Students were viewing live video from the robot’s camera displayed on their computer, and through this image they tele-operated the robot from their computer keyboard. To improve the movements of the robot in the room, they also used distance measurement and a simple obstacle avoidance Python script. 

#### 4.2.2. Line Following Using the Camera

The goal of this project was to use the camera as a color sensor in order to direct EUROPA to follow a yellow line painted on the floor. At the beginning of the lesson, the principles of digital vision sensors were explained to students and a reference was made to RGB color space. A simple experiment with the camera and a simple user interface based on OpenCV demonstrates how object colors are transformed in RGB values, using the camera. Then, HSV color space was introduced and an explanation was given as to why it is best to use HSV in conditions of unstable luminosity. A simple line-following algorithm based on color detection was presented and was applied in a Python script based on OpenCV. The program measures deviations from the yellow line measured in pixels and transforms them into appropriate wheel speeds for the differential drive. The algorithm is robust and results in smooth line following, better than using the infrared sensor commonly applied to this kind of experiment.

#### 4.2.3. Simultaneous Localization and Mapping

The last experiment introduces the advanced topic of Simultaneous Localization and Mapping (SLAM). At the beginning of the lesson, a reference was made to how the LIDAR works, and the students saw a point cloud in rviz, representing the distances from the obstacles in the room. Subsequently, reference was made to mapping and its importance in robotics. Finally, the concept of Bayesian update using sensor measurements was introduced in general terms and a connection was made to similar concepts taught in mathematics lessons on probabilities. The Hector SLAM function [65] was introduced and students saw the mapping of their classroom in rviz.

### 4.3. Performance Evaluation Experiments

#### 4.3.1. Odometry Evaluation

**Figure 4:**
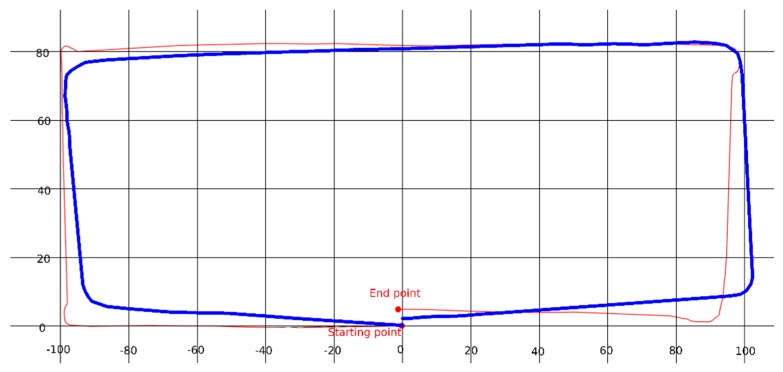
Evaluation of odometry measurements using the optical encoders.

During this test, we commanded the robot to traverse a predefined orthogonal path with dimensions 0.8 m by 2 m and return to its original position. In Figure 4, the performed path is shown in red. The robot follows the commands quite accurately, with a most notable deviation from the commanded path observed during the final stages of the route. Although the robot was commanded to end up exactly at its starting point, the difference between the end point and the starting position is 3 cm in the horizontal axis and 9 cm in the vertical one. The blue line corresponds to the odometry as perceived by the robot. Odometry was measured using optical encoders. The axes units in the figure correspond to cm.

#### 4.3.2. Mapping

Hector-SLAM is a ROS package that is able to solve the robot localization and mapping problem for a 6DOF vehicle equipped with a laser scanning system (LIDAR) and inertial sensors [66]. The package fuses 3D robot attitude and position information obtained from an Inertial Measurement Unit with a 2D SLAM process. The SLAM process in Hector-SLAM is based on occupancy grid mapping combined with 2D pose estimation. At each step, the system aligns the new laser scan endpoints with the map learned so far. The optimization of the alignment process results in an estimate of the new position of the robot in the 2D map. In this way, the environmental map and the robot pose are produced incrementally, starting from a known pose.

In our EUROPA robot, the movement is on a plane and only the position (x, y) and orientation ψ on the plane is relevant. Therefore, only the 2D SLAM process is active and IMU information is not required. We have built a model environment, with approximate dimensions 2 m × 2 m, which can be traversed by the robot, starting from a known initial position and completing full circles around a corridor. At each step, the laser scanner acquires a cloud of points from the surrounding walls and computes the new change in translation and orientation, based on a transformation that gives the best alignment with the previous map. Knowing the new pose, the occupancy grid is updated. 

**Figure 5:**
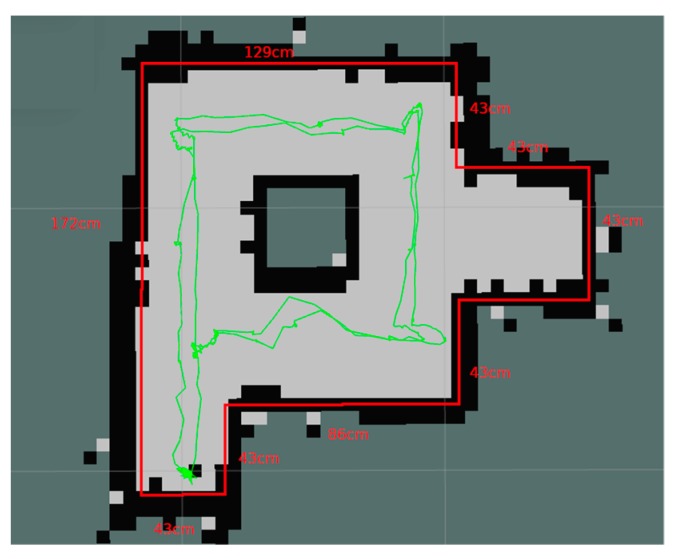
Evaluation of the robot’s ability to create maps.

In Figure 5 the environmental map created by the Hector-SLAM process is presented. Occupied cells are shown in black, while lighter color represents empty space. The red line illustrates the ground truth information of the model environment. The green line is the pose as it is computed during the SLAM process. The mapping was created after two loops around the corridor and the vehicle ended at its starting position. The Hector-SLAM package does not require odometry from optical encoders as input in the process. 

The origin of the inertial frame is considered to coincide with the starting point of the robot track. The map was generated by teleoperating the robot with linear velocities less than 0.5 m/sec and angular velocities less than 0.314 rad/sec. Both the map and the pose estimation are considered to be satisfactory for the educational purpose served by our experiment. 

#### 4.3.3. Line Following Performance

**Figure 6:**
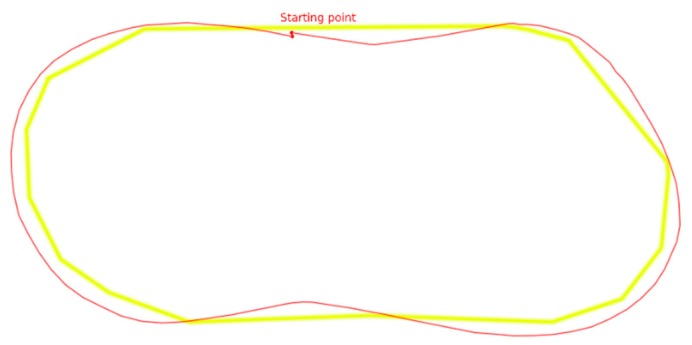
Line-following setup—The yellow line is the target path, the red line is the true path.

In order to assess the performance of the line following problem, we have created a yellow curved path of a total of 4.2 m, as in Figure 6. 

**Figure 7:**
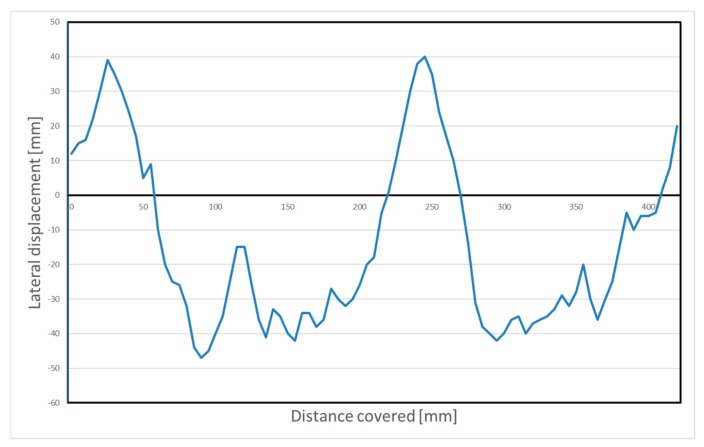
Line-following—displacement of the robot related to the line.

The actual robot path is shown by the red line. In Figure 7, the displacement of the robot from the center of the yellow line is shown, measured in mm. In this figure, the horizontal axis is the distance covered by the robot. At the starting point the robot was not in the center of the yellow line and had a 10-degree clockwise rotation. The line-following algorithm extracted color features from the image frame captured by the camera, using the OpenCV library. A simple P-controller was selected in order to direct the robot across its path. The P-controller was selected for educational reasons. The oscillations observed in Figure 7 are mainly due to the simplicity of the controller.

### 4.4. Assessment of EUROPA in the Classroom

The target group for the assessment was two second-grade high school classes or 22 and 23 students in Greece of ages 16 to 17. The lessons were performed as a part of a series of technology projects that have been added into the Greek curriculum during the past few years and correspond to a weekly workload of 2 h. The main goal of EUROPA in these technology projects was to provide students with real world science examples and a better understanding of notions that they have already been taught in lessons such as physics, mathematics and computer science. The students were acquainted with more advanced technological subjects and were motivated for independent learning and discovery. The acceptance of the platform was enthusiastic. All students were able to follow, understand and work on the EUROPA robots without any serious problems and some of them were even willing to drill down to the robot’s architecture. 

Regarding physics and mathematics, EUROPA’s impact was evident since among others, students had a chance to relate theoretical kinematics and dynamics with practical robot movement. They saw that the distribution of mass in an object can affect its movement. They applied theoretical knowledge on rotational movement to wheel rotation and connected it to odometry calculations. They also had a chance to apply trigonometry and vector analysis to real world problems. EUROPA also proved to be a great medium for the introduction of students to new concepts such as sensors, actuators, control, and physical computing. In programming lessons, the students applied programming skills in solving real problems, which gave them a totally new incentive for writing code and understanding programming structures. They faced the notion that hardware abstraction and standards are particularly important in order to make sensory information usable and that working in simulation is quite different than working in the real world.

At the end of the semester, the students had clearly a better understanding of real-world problems solved by science, and their interest in technology was higher than with a similar course designed with a LEGO-like platform. The openness of the platform and the advanced scenarios that were shown to the children proved to be quite important to motivate them.

## 5. Conclusions 

This paper reviews existing educational robot platforms for various levels of education, from kindergarten to university. Our research reveals that there is a gap in the low-cost range of ROS-based educational robotics. However, ROS-based robotics is versatile and has great potential for integration with free simulation and visualization software, as well as with advanced sensors. A ROS-based, low-cost platform can support advanced projects, like machine vision, machine intelligence, localization and mapping. This gave us the incentive to build EUROPA which is a cheap and versatile open platform based on ROS. It can cover a range of applications, from basic educational robotics to advanced applications, such as vision and mapping. Its main controller is the Raspberry Pi, which is supported by a great community and can readily use a plethora of applications. The platform is currently being assessed in two secondary schools in Central Macedonia, Greece, under a pilot robotics curriculum. Future work includes redesigning both the platform and the curriculum, after receiving feedback from pilot schools. We also aim to build an online community, supporting students and teachers with educational material and extensive documentation. 

## Figures and Tables

**Table 1:** Most widespread mobile educational platforms: target group, cost and technology.

Bot Name	Level of Education	Appro-ximate Cost (€)	Open Source HW/SW	Sensors	Actuators/ Kinematic Model	ROS	Controller/CPU	Programming Tools	Data Communication	Ref
Bee-bot /Colby	Kindergarten/Elementary	65	NO/NO	Bs	?/DD	NO	?	Buttons	NO	[26,27]
mBot	Elementary/ secondary	120	NO/YES	B, UDS, LF, LS, IR	DC gear motor plastic, RGB LEDs, buzzer/DD	NO	ATmega328/ motor driver	Block-based/ Arduino IDE	BT	[44]
Thymio II	Elementary/ secondary	170	YES/YES	Bs, IR, LF, Th, Acc, Mic	DC gear motor plastic, LEDs, Loud speaker/ DD	NO	PIC24FJ128GB/ motor driver	Block/Visual /text based (Aseba)	2.4 GHz, protocol 802.15.4	[28,29]
Edison	Elementary/ secondary	60	NO/NO	Bs, IR, LS, LF, Mic, OE	DC gear motor, LEDs, buzzer/DD	NO	Freescale 8-bit MC9S08PA16	Block-based/ Scratch/EdPy	IR	[46]
Scribbler 3	Elementary/ secondary	200	NO/NO	LS, LF, IR, OE	DC gear motor, LEDs/DD	NO	Propeller P8X32A	Block-based programming	USB	[30]
LEGO EV3	secondary	500	NO/NO	TS, CS, GS, UDS	Compact Gear motors/DD	NO	ARM9	EV3 icon-based software	BT, Wi-Fi	[32]
AlphaBot2	secondary	90–125	YES/YES	UDS, IR, LF, Camera*	N20 micro gear motor, RGB LEDs/DD	NO	Arduino or Raspberry Pi zero or BBC micro:bit	Arduino IDE or Python	BT, IR	[47]
ActivityBot	secondary	200	NO/NO	UDS, LS, TS, IR, OE	High speed 360^o^ servos/DD	NO	Propeller P8X32A	Block-based graphical/C	USB	[30]
Epuck 2	Secondary/ higher	1200	YES/YES	IR, acc, gyro, mic, camera, ToF	Stepper motors, LEDs, Loud-seaker/DD	YES	STM32F4 ARM Cortex M4	Free C compiler	BT	[33,34]
Robobo	Secondary/ higher	450	NO/YES	Camera, acc, gyro, GPS, magn, IR, LS	DC gear motor, LEDs/DD	YES	Smartphone + PIC32 (low-level control)	Scratch/ Python/ ROS	Wi-Fi	[48,49,50]
EUROPA II	Secondary/ higher	120 or 300**	YES/YES	UDS, IR, OE, Camera, LIDAR**	DC gear motor, robotic arm, LEDs/DD	YES	Raspberry Pi	Python/ROS/ OpenCV	Wi-Fi	[51]
Turtlebot 3 (burger)	higher	800	YES/YES	Camera, LIDAR, acc, gyro, magn	DYNAMIXEL AX gear motor + driver/DD	YES	Raspberry Pi + OpenCR	Block-based/ ROS/Python	Wi-Fi	[35,52]
Duckiebot	higher	150	YES/YES	Fish-eye camera for Raspberry Pi	DC gear motor/DD	YES	Raspberry Pi 2	ROS programming in C/Python	Wi-Fi	[13,36]
Leo Rover	Research/ industry	2500	YES/YES	Fish-eye camera, wheel encoders	4x DC gear motor	YES	Raspberry Pi + Core2 ROS (low level)	ROS Programming	Wi-Fi	[38]
Summit-XL	Industry	15000	YES/YES	3D camera, Laser scanner + optional sensors	4x DC gear motor/skid steering	YES	Intel processor/PC	ROS Programming	Wi-Fi	[39]

?: Not documented, DD: Differential Drive, B (s): Button (s), UDS: Ultrasonic Distance Sensor, LF: Line Following sensor, LS: Light Sensor, IR: Infrared Proximity Sensor, TS: Touch sensor, CS: Color sensor, Acc: accelerometer, Mic: Microphone, OE: Optical Encoder, ToF: Time of Flight distance sensor, magn: magnetometer, BT: Bluetooth *: in RPi version, **: University Edition.

**Table 2:** List of EUROPA parts and approximate cost.

Part	Number	Cost in Euros
Raspberry 3 B+ board	1	41.90
Motor controller shield DRV8833	1	5.20
Raspberry Pi camera	1	21.20
Servo motors Feetech FS90	3	3 × 2.5 = 7.50
Led photodiode encoders	2	2 × 3.90 = 7.80
Ultrasonic sensor	1	2.50
DC motors with encoder disks	2	2 × 1.8 = 3.60
Plexiglas double base	1	1.80
Caster ball	1	1.60
Rechargeable battery	1	9.90
Wheels	2	2 × 1.5 = 3.00
Cables		4.00
Lidar	1	180.9
mini pan tilt kits	2	2 × 2.9
robotic arm axles (3d printed)	2	0.5
Spacers, bolts, nuts		2.0
Total Cost		299.2
